# Nitrogen and phosphorus removal from anaerobically digested wastewater by microalgae cultured in a novel membrane photobioreactor

**DOI:** 10.1186/s13068-018-1190-0

**Published:** 2018-07-09

**Authors:** Xi Chen, Zhipeng Li, Ning He, Yanmei Zheng, Heng Li, Haitao Wang, Yuanpeng Wang, Yinghua Lu, Qingbiao Li, YaJuan Peng

**Affiliations:** 1 Department of Chemical and Biochemical Engineering, College of Chemistry and Chemical Engineering, The Key Lab for Synthetic Biotechnology of Xiamen City, Xiamen University, Xiamen, 361005 People’s Republic of China; 2 College of Food and Biological Engineering, Jimei University, Xiamen, People’s Republic of China

**Keywords:** MPBR, ADW, Suspended solids, Membrane pore size, Multi-batch, Cultivations

## Abstract

**Background:**

With the further development of anaerobic digestion, an increasing output of anaerobically digested wastewater (ADW), which typically contained high concentrations of ammonium, phosphate, and suspended solids, was inevitable. Microalgae cultivation offered a potential waste-to-value strategy to reduce the high nutrient content in ADW and obtain high value-added microalgae. However, ADW generally contained a mass of pollutants (suspended solids, competitors, etc.), which could inhibit microalgae growth and even result in microalgae death by limiting light utilization. Thus, it is highly imperative to solve the problem by a novel modified photobioreactor for further practical applications.

**Results:**

Four microalgae species, *Scenedesmus dimorphus*, *Scenedesmus quadricauda*, *Chlorella sorokiniana*, and *Chlorella vulgaris* ESP-6, were cultivated in the membrane photobioreactor (MPBR) fed with ADW to investigate the efficiency of ammonia and phosphorus removal. The results showed that *C. sorokiniana* had the best performance for the removal of ammonia and phosphorus from ADW. The highest amount of *C. sorokiniana* biomass was 1.15 g/L, and the removal efficiency of phosphate (66.2%) peaked at an ammonia concentration of 128.5 mg/L after 9 days’ incubation. Moreover, the MPBR with 0.1 μm membrane pore size had the best ammonia and phosphate removal efficiencies (43.9 and 64.9%) at an ammonia concentration of 128.5 mg/L during 9 days’ incubation. Finally, the continuous multi-batch cultivation of *C. sorokiniana* was performed for 45 days in MPBR, and higher removal ammonia amount (18.1 mg/day) and proteins content (45.6%) were obtained than those (14.5 mg/day and 37.4%) in an normal photobioreactor.

**Conclusion:**

In this study, a novel MPBR not only eliminated the inhibitory effects of suspended solid and microorganisms, but also maintained a high microalgae concentration to obtain a high amount of ammonia and phosphate removal. The research provided a theoretical foundation for the practical application of MPBRs in various wastewater treatment schemes without pretreatment by algae, which could be used as biofuels or protein feed.

## Background

Anaerobic digestion was considered to be a promising option for the treatment of waste, which could solve the problem of waste contamination and produce energy. With the further development of anaerobic digestion, an increasing output of ADW, which typically contained high concentrations of ammonium, phosphate, and suspended solids, was inevitable and approximately 385 million tons of liquid waste had been generated by over 30 million methane-generating tanks [1]. ADW also had a high N:P ratio and chemical oxygen demand (COD), so it was difficult to treat ADW by conventional biological processes, such as by waste lagoons [2], sequencing batch biofilm reactors (SBBRs) and sequencing batch reactors (SBRs) [3], which was complex, had a higher cost or did not have renewable matter cycles during treatment.

Microalgae cultivation offered a potential waste-to-value strategy to reduce the high nutrients content in ADW and obtain high value-added microalgae [4]. Although several freshwater microalgae species had already been applied to reduce the nutrient concentration in wastewater and produce lipids for biofuels [5], reports of wastewater, especially ADW, treatment by microalgae cultivation was still limited. The main reason was that ADW generally contained a mass of pollutants (suspended solids, competitors, etc.), which could inhibit microalgae growth and even result in microalgae death by limiting light utilization. Thus, UV radiation, membrane filtration, centrifugation, and autoclaved sterilization had been used as a pretreatment methods before ADW was applied as the culture medium for the cultivation of microalgae [6]. However, these processes had numerous disadvantages, including costliness, instability or complexity, and so on [7].

**Fig. 1:**
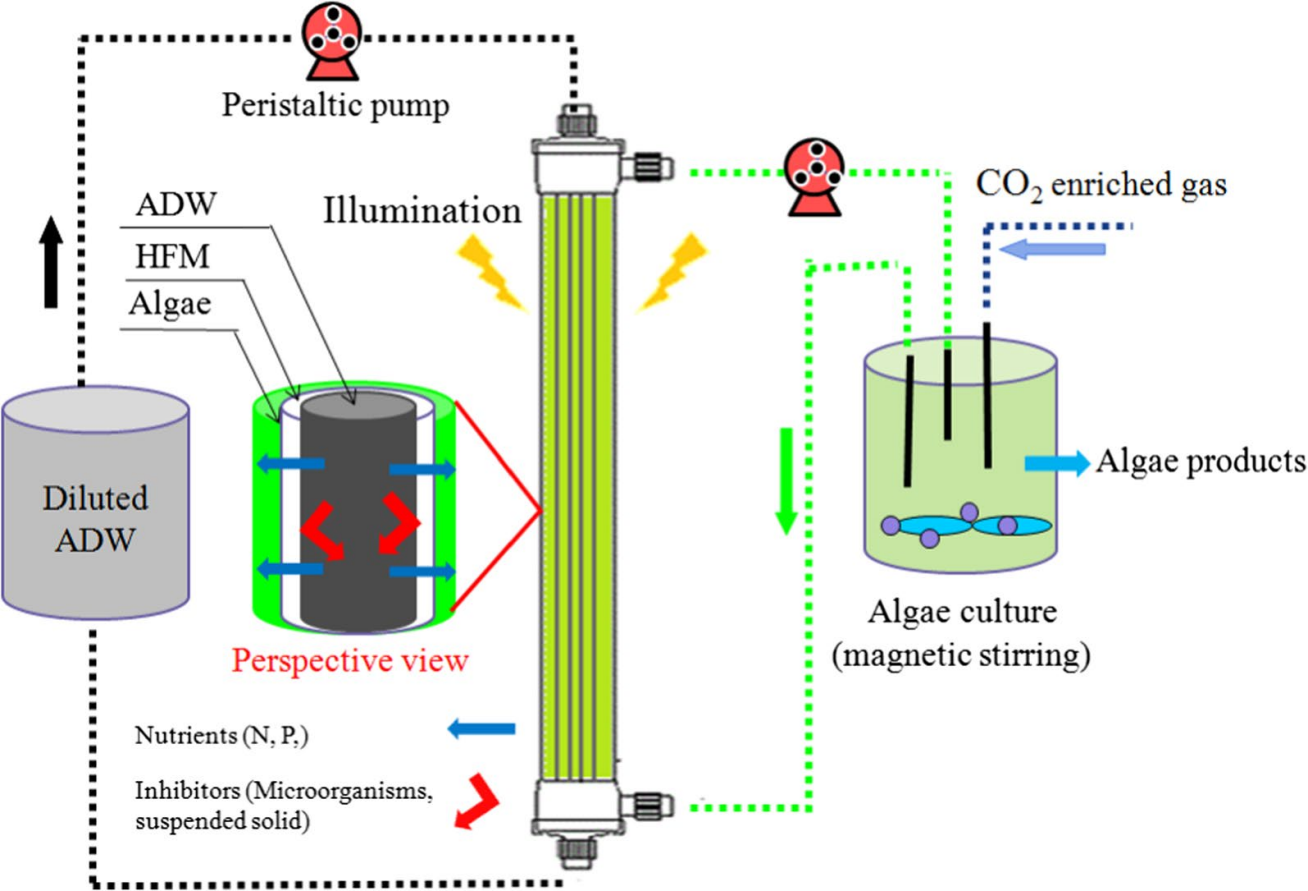
The schematic diagram of membrane photobioreactor (MPBR)

To solve the problem, a MPBR with the introduction of hollow fiber membranes (HFM) was proposed for the exploitation of nutrients in untreated ADW by algae (Fig. 1), in which the nutrients could permeate from the ADW in the inner chamber to the microalgae culture medium in the outer chamber through the HFM for microalgae growth, but the pollutants in ADW that inhibited microalgae growth (suspended solids, competitors, etc.) could not directly contact the microalgae cells by membrane interception, and thus, the negative effects caused by ADW could be substantially eliminated. Although some studies had investigated the application of membrane in photobioreactors [8, 9], similar to membrane bioreactor, the membranes were only used for increasing microalgae concentration by filtration through the separation of algae and water, which had no significant effect on eliminating pollutants (suspended solids, competitors, etc.) that inhibited microalgae growth from ADW.

Herein, a novel submerged membrane permeation system in a photobioreactor was designed for ADW treatment with microalgae, and the performance of the MPBR during continuous multi-batches cultivations was also evaluated. The results showed that the biomass production of microalgae and the efficiency of nutrients removal from ADW in MPBR were much higher than in NPBR. The novel MPBR demonstrated a potential approach for microalgae cultivation using various wastewaters without pretreatment. In addition, the research also provided some valuable foundations for the practical application of MPBRs in wastewater treatment by algae.

## Results and discussion

### Different microalgaes cultivated with ADW in MPBR and NPBR

**Fig. 2:**
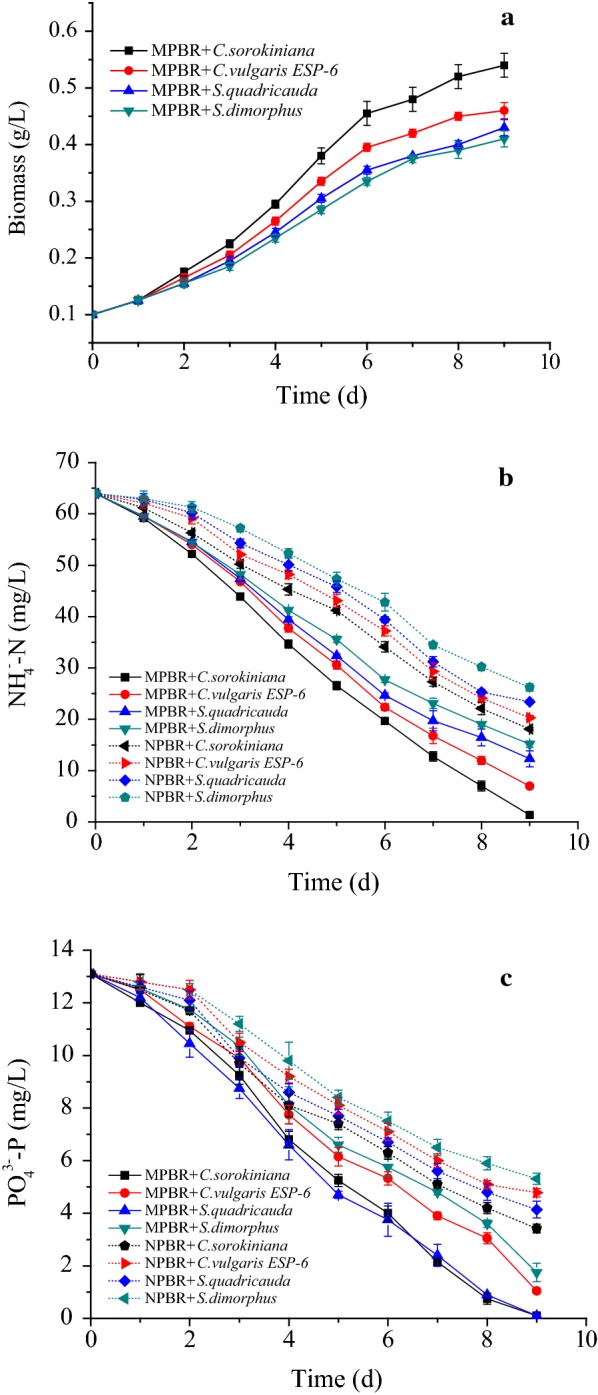
Time-course records of **a** biomass, **b** ammonia concentration and **c** phosphate concentration for four algaes in diluted ADW in MPBR and NPBR

Four microalgae species (*S. dimorphus*, *S. quadricauda*, *C. sorokiniana*, and *C. vulgaris ESP*-*6*) were selected to assess the performance of microalgae growth and nutrients uptake in diluted ADW (64.3 mg/L ammonia, 13.1 mg/L phosphate, 0.86 g/L suspended solids) in MPBR and NPBR. The results shown in Fig. 2a indicated that the biomass of all microalgae increased in the MPBR in 9 days’ incubation. All four algae species removed over 75% of the ammonia nitrogen from the MPBR after 9 days of incubation. However, the amount of ammonia and phosphate removed by the four algae species from the NPBR was lower than that removed from the MPBR due to the pollutants (suspended solids, competitors, etc.) in ADW inhibiting microalgae growth (Fig. 2b, c). Over all, *C. sorokiniana* had the best performance, with an ammonia removal efficiency of 97.9% and a biomass concentration of 0.54 g/L in the MPBR. In addition, phosphate was absorbed more quickly by *C. sorokiniana* and *S. quadricauda* (99.2 and 98.5%) in the MPBR.

*Chlorella sorokiniana* has been reported to grow at higher growth rates and has the potential for high-density cultivation in wastewater [10, 11]. De-Bashan et al. [12] also demonstrated that *C. sorokiniana* was able to grow in wastewaters under conditions that were unfavorable for other algal species. Thus, *C. sorokiniana* was chosen for further study.

### MPBR performances under different conditions

Properties of a MPBR greatly varied with different conditions, and thus had different impacts on microalgae growth. To investigate the applicability of the proposed MPBR, ADW with different characteristics (i.e., ammonia and suspended solids concentration) and membrane pore size were utilized to investigate the performance of MPBR in the following sections.

#### Ammonia concentration of ADW

It was demonstrated that the nitrogen limitation [13] or excessive nitrogen source [14] could reduce the growth rate and productivity of microalgae. However, the initial concentration of ammonia nitrogen in ADW was distinctly high (1000–1200 mg/L), so moderate ammonia concentration should be investigated.

**Fig. 3:**
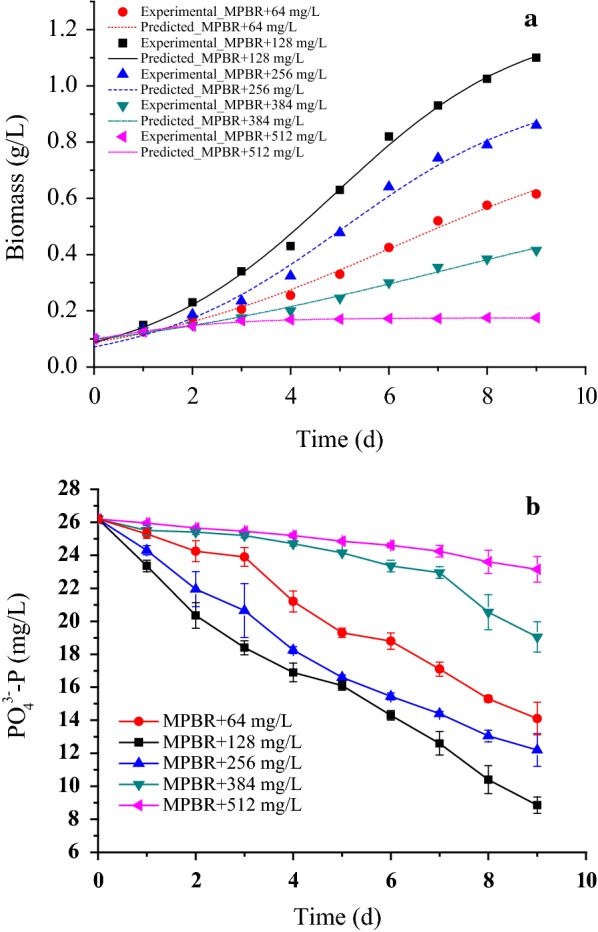
Influence of ammonia concentration on **a** biomass production and **b** phosphate removal by *C. sorokiniana* in MPBR. In the figure of biomass production, symbols represent experimental data and lines represent the fitted line by modified logistic mode

The initial concentration of ammonia nitrogen in ADW was diluted to 64.3 (6.2%), 128.5 (12.5%), 257.0 (25%), 385.5 (37.5%), and 514.0 mg/L (50%). A comparative study was done with respect to the growth profile of *C. sorokiniana* in the MPBR (Fig. 3a). The algae biomass was positively correlated with the ammonia concentrations at levels lower than 128.5 mg/L. The highest algae biomass was 1.15 g/L at an ammonia concentration of 128.5 mg/L. However, the algae biomass dropped from 0.86 g/L with 257.0 mg/L ammonia to 0.16 g/L with 514.0 mg/L ammonia.

A logistic model was found to fit the experimental biomass data obtained at different ammonia concentrations well (Table 1). The maximum biomass concentration (*X*_max_ = 1.236 g/L) and apparent specific growth rate (*K*_c_ = 0.523 day^−1^) were significantly higher at 128 mg/L ammonia than at the other concentrations. *X*_max_ indicated the carrying capacity of *C. sorokiniana* which could be defined as the maximum algal biomass concentration that could be supported under particular experimental conditions considering the impact of different ammonia concentrations on the algal population. The curve fitting was found to be in good agreement with the experimental data, as the *R*^2^ was 0.997.

**Table 1:** Simulated logistic model for microalgae growth and phosphate absorption rate (PAR) at different ammonia concentrations

	NH_4_^+^-N (mg/L)				
	64.3	128.5	257.0	385.5	514.0
*X*_0_ (g/L)	0.088	0.088	0.071	0.097	0.099
*X*_max_ (g/L)	0.857	1.236	0.978	0.687	0.175
*K*_c_ (day^−1^)	0.355	0.523	0.503	0.253	0.713
*R* ^2^	0.992	0.997	0.991	0.991	0.991
PAR (mg/g day)	1.73	2.82	2.41	0.71	0.31

The removal efficiency of phosphate peaked at 66.2% at an ammonia concentration of 128.5 mg/L (Fig. 3b), at which the phosphate absorption rate (PAR) was also higher (2.82 ± 0.2 mg/g day), as shown in Table 1. The phosphate removal efficiency dropped from 53.4 to 11.6% when the concentration of ammonia was 257 and 514 mg/L.

The growth of *C. sorokiniana* cultivated in different diluted concentrations of ADW was affected by the nutrients in the diluted ADW. Because of insufficient nutrients in 6.2% (V/V) diluted ADW, the biomass of *C. sorokiniana* increased only slightly. In this research, 25% (V/V) diluted ADW contained roughly 257.0 mg/L ammonia nitrogen, and some studies had demonstrated this ammonia concentration could exert inhibitory or toxic effects on algae growth [15]. The increasing ammonia concentration in 37.5% (V/V) and 50% (V/V) diluted ADW might further exceed the toleration limit of microalgae, thus the inhibition on microalgal growth was further strengthened. A proper ammonia concentration in 12.5% (V/V) (128.5 mg/L) could activate some enzymes such as nitrate and nitrite reductase [16] and enhance the synthesis of macromolecules such proteins and chlorophyll [17]. Similar results had been reported in other investigations on microalgae cultivation in wastewater [18], where it was observed that relatively lower wastewater dilution ratios decreased algae growth rates and decreased the removal efficiency of phosphate by algae due to inhibition by the high ammonia concentration in wastewater.

#### Suspended solids in ADW

High turbidity is a typical characteristic for most wastewaters. The suspended solids in ADW, which was not easily isolated, could severely affect the penetration of light, leading to microalgae growth inhibition. However, the introduction of HFM in MPBR could effectively solve the problem by insulating the suspended solids in the inner chamber of the MPBR.

**Fig. 4:**
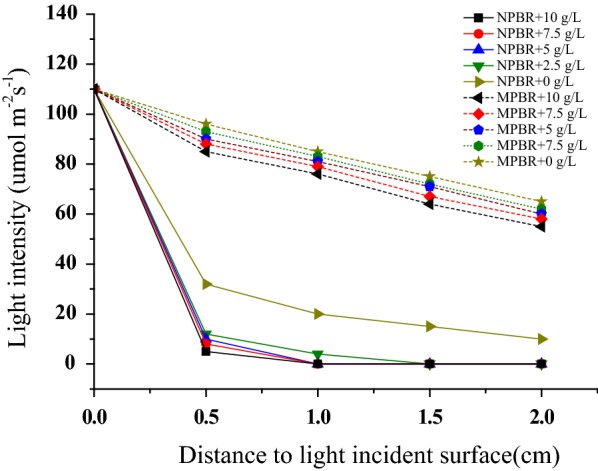
Variation of light penetration between the MPBR and the NPBR with ADW at different concentrations of suspended solids

Figure 4 shows the light distribution in the cultures of MPBR and NPBR with ADW at different concentrations of suspended solids (pure system of ADW without algae cultivation). It could be observed that 59.2% of the incident light could transmit across the culture medium in MPBR, whereas only 9% of incident light transmitted across the ADW in NPBR at the initial stage of 0 g/L suspended solids concentration. Thereafter, along with the increase of suspended solids concentration, the light intensity in the culture of the NPBR at a distance of 1 cm from light incident surface had dropped constantly to almost zero. However, compared with the NPBR, there was more light distributed in the microalgae culture of the MPBR at 10 g/L suspended solid since the light shading effect caused by suspended solids was avoided, plenty of light (76 μmol^−2^ m^−1^) still existed at a distance of 1 cm from light incident surface in the microalgae culture in MPBR since the light shading effect caused by suspended solids was avoided (Fig. 4).

**Fig. 5:**
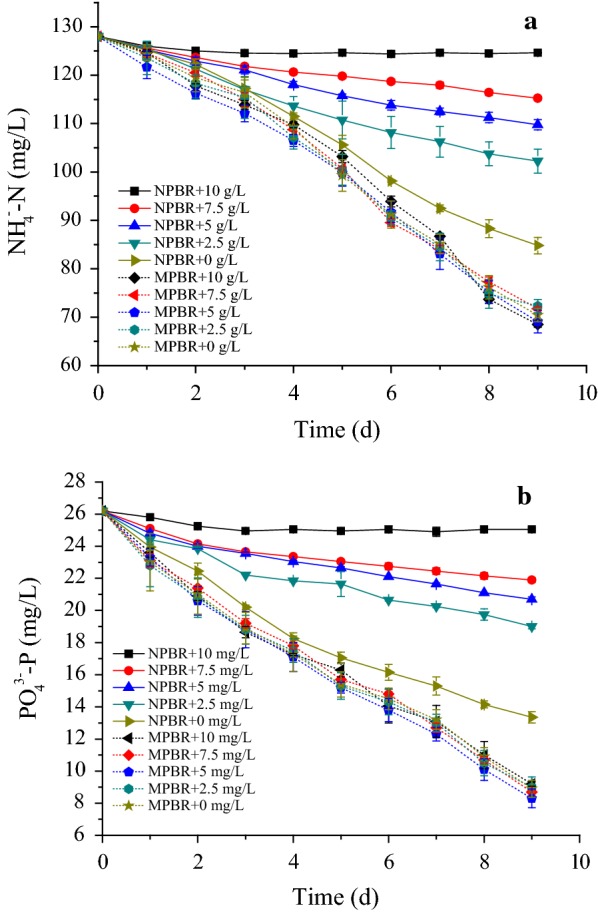
Effect of suspended solids on **a** nitrogen and **b** phosphate removal by *C. sorokiniana* in MPBR and NPBR

As a result, the removal efficiencies of ammonia and phosphate decreased with the increased suspended solid concentration in NPBR (Fig. 5). The removal ammonia amount decreased from 86.5 to 6.7 mg/L, and the removal phosphate amount dropped from 25.7 to 2.3 mg/L when the concentration of suspended solid increased from 0 to 10 g/L (Fig. 5a, b). Although the light in NPBR was mostly shaded by the suspended solids, the existence of organic matter in ADW provided organic carbon source for microalgae growth. It convinced that the light attenuation effects in NPBR had become the major inhibitory factor for microalgae growth after COD exhaustion [19]. However, in MPBR, there was no obvious inhibitory effect on microalgae growth, even when the concentration of suspended solids increased to 10 g/L. The removal ammonia and phosphate amounts reached 115.5 and 34.7 mg/L, respectively. The results demonstrated that the light attenuation effects caused by suspended solids were significantly reduced in MPBR.

### Effect of membrane pore size in the MPBR

The effect of membrane pore size on the performance of the MPBR, which played a important role in eliminating the inhibition of microalgae growth due to suspended solids and competitors in the ADW, was investigated in this study.

**Fig. 6:**
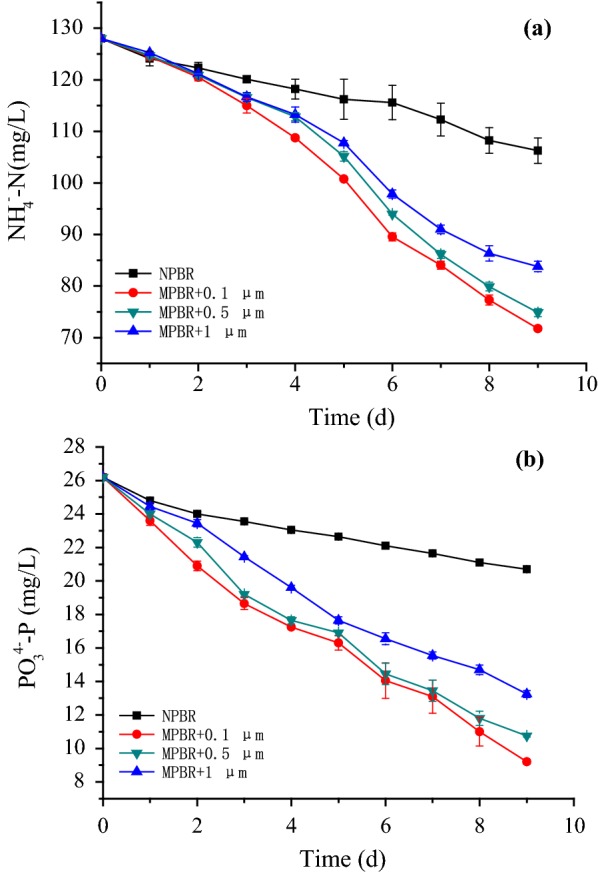
Effect of different membrane pore size on **a** nitrogen and **b** phosphate removal by *C. sorokiniana* in MPBR

The initial membrane pore sizes of the MPBR were 0.1, 0.5, and 1 μm. *C. sorokiniana* cultivated directly in the PBR containing ADW with 5 g/L suspended solids was taken as the control. When the membrane pore size was enlarged, the ammonia and phosphate removal efficiencies by *C. sorokiniana* declined constantly in Fig. 6. The ammonia removal efficiency decreased from 43.9 to 34.5% (Fig. 6a), and the phosphate removal efficiency dropped from 64.9 to 49.2% (Fig. 6b), when the membrane pore size increased from 0.1 to 1 μm. However, the ammonia and phosphate removal efficiencies in the NPBR dropped the most, reached only 20.1 and 21%, respectively (Fig. 6a, b).

Based on microfiltration membrane permeation experiments of ADW and deionized water, when the nanoscale particle size of ammonia and phosphate was much smaller than the microscale membrane pore size, the penetration rates of ammonia and phosphate had no significant difference in different membrane pore sizes. However, the penetration rate of microscale competitors and suspended solids increased constantly with as the enlarged membrane pore size [20, 21]. From the above results, it determined that the light shading effect caused by suspended solids in the ADW was key factors harming the growth of *C. sorokiniana*. And the light intensity was the driving force for microalgae growth and metabolism. Insufficient light could severely limit the growth of microalgae [19]. Besides, more competitors would also inhibit microalgae growth. Hence, the ammonia and phosphate removal efficiencies of *C. sorokiniana* decreased with enlarged membrane pore size. Moreover, *C. sorokiniana* came in direct contact with suspended solids in the ADW in NPBR, so the nutrients removal efficiencies declined in the most.

### Multi-batch cultivation of *C. sorokiniana* with ADW in MPBR and NPBR

To evaluate the overall performance between the MPBR and NPBR, the continuous multi-batch cultivation was conducted under the optimum parameters (128.5 mg/L ammonia, 26.1 mg/L phosphate, and 0.1 μm membrane pore size).

**Fig. 7:**
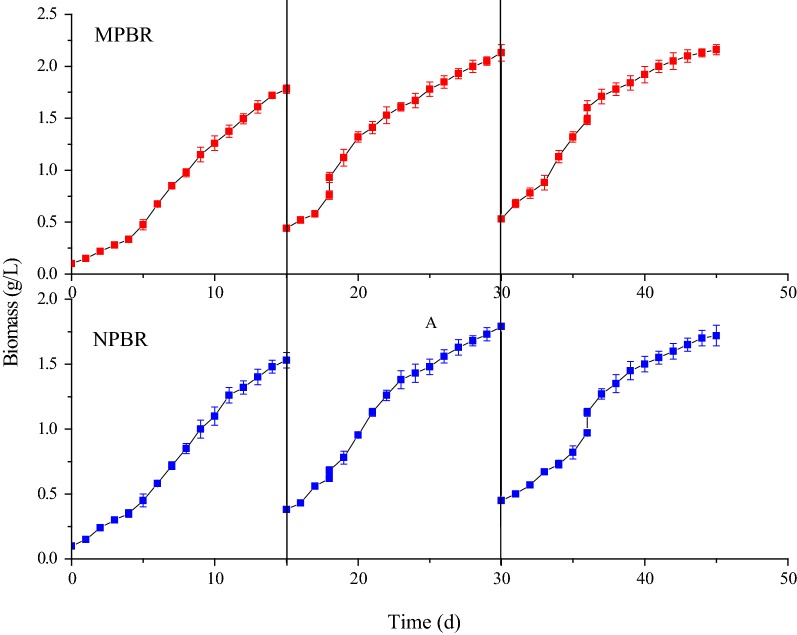
Time-course record of biomass during multi-batch cultivations of *C. sorokiniana* in MPBR and NPBR

From Fig. 7, *C. sorokiniana* had a biomass growth of 1.69 g/L, and the average specific growth rate was 0.085 day^−1^ during the second batch of 15 days, and the microalgae biomass in the third batch had no obvious growth in MPBR. The possible reason was that the light attenuation caused by self-shading effect had become the predominant limiting factor for microalgae growth. However, *C. sorokiniana* had a lower biomass growth of 1.41 g/L in NPBR than that in MPBR.

A logistic model was found to fit well with the experimental biomass data obtained between MPBR and NPBR (Table 2). When the operation of MPBR went into stable state, the maximum biomass concentration (*X*_max_) and apparent specific growth rate (*K*_c_) showed little difference during 2nd and 3rd batch of multi-batch cultivation. Compared to multi-batch cultivation in NPBR, the *X*_max_ (2.188 g/L) and *K*_c_ (0.299 day^−1^) of 3rd batch cultivation in the MPBR were higher.

**Table 2:** Simulated logistic model for algae growth between MPBR and NPBR

	MPBR			NPBR		
	1st	2nd	3rd	1st	2nd	3rd
*X*_0_ (g/L)	0.112	0.425	0.526	0.116	0.424	0.393
*X*_max_ (g/L)	1.898	2.151	2.188	1.645	1.892	1.858
*K*_c_ (day^−1^)	0.350	0.283	0.299	0.329	0.259	0.247
*R* ^2^	0.997	0.993	0.996	0.999	0.991	0.995

As shown in Table 3, the average daily removal amount of ammonia and phosphate (18.1 and 3.63 mg/day, respectively) by *C. sorokiniana* in the MPBR was higher than that (14.5 and 2.76 mg/day, respectively) in the NPBR. If the suspended solids of ADW had not been centrifuged out from NPBR, the removal amount difference of ammonia and phosphate between MPBR and NPBR would be more significant. The nutrients absorption difference between MPBR and NPBR may result from pollutants in ADW eliminated by MPBR that promoted microalgae growth to assimilate more ammonia and phosphate in MPBR.

**Table 3:** The growth differences of *C. sorokiniana* between multi-batch cultivation of NPBR and MPBR

	Biomass	NH_4_-N		PO_4_-P		Protein (%)
	Growth amount (g/L)	Removal rate (mg/g day)	Removal amount (mg/day)	Removal rate (mg/g day)	Removal amount (mg/day)	
NPBR	1.41 ± 0.1	13.2 ± 0.7	14.5 ± 0.4	2.65 ± 0.2	2.76 ± 0.2	37.4 ± 1.1
MPBR	1.69 ± 0.1	13.5 ± 0.5	18.1 ± 0.6	2.71 ± 0.2	3.63 ± 0.3	45.6 ± 1.7

In general, a fraction of microalgae biomass flew away with discharged treated ADW when the nutrients in ADW are exhausted in batch cultivation in NPBR [22], in which microalgae and ADW mixed completely. Because of reduced microalgae concentration, the removal amount of ammonia and phosphate by microalgae would decrease for ADW treatment during the preliminary stage of next batch cultivation. However, only treated ADW was replaced with raw ADW when the nutrients were exhausted in multi-batch cultivation in MPBR, and the microalgal biomass would not flow out with the discharged ADW to maintain high microalgal concentration, so that the removal amount of ammonia and phosphate in MPBR would be much higher than that in NPBR, and the treatment efficiency of ADW would also be increased in MPBR.

The content of proteins between multi-batch cultivation of NPBR and MPBR is shown in Table 3. Compared with that (37.4%) in NPBR, the protein content of *C. sorokiniana* was higher (45.6%) in MPBR.

Because the medium had enough nutrients for the algae growth at the preliminary stage of algae cultivation in batch cultivation in NPBR, the algae could accumulate protein in their cells well. However, at the later stage of algae cultivation, the nutrients of medium were depleted by algae, so the algae had to assimilate autologous protein to maintain autologous growth [23], which resulted in a decline in the protein content [24].

By comparison of algae protein contents in NPBR and MPBR, the treated ADW replaced with supplemented raw ADW in every batch cultivation could continue to provide nutrients for algae growth in MPBR, so the MPBR was suitable for proteins accumulation in algae. Although the protein contents of *C. sorokiniana* was not particularly prominent, to some species of protein-rich microalgae, the technology of continuous multi-batch cultivation in MPBR may provide a feasible access to further improve its proteins content.

With MPBR, the pretreatment of wastewater and pre-acclimation of microalgae strains could be avoided, which substantially reduced the energy cost, and investment on freshwater and commercial fertilizers. However, membrane fouling is a common problem in wastewater treatment reactor. In general, membrane could be regenerated with ultrasonic cleaning, hydraulics flushing [25], alkaline, and oxidant cleaning [26]. In MPBR, less suspended solid and algae aggregated on the membrane surface, so that could be easily removed with ultrasonic cleaning and back flush, ensuring that the MPBR could be reused for microalgae cultivation for more than 2 months.

Algal biomass has been widely regarded as one of the most promising raw materials to compensate the increasing global demand for food, feed, and biofuels and chemical production [27, 28]. Reutilization and refinery of microalgae are also hot topics in recent years. Various biofuels were reported to be produced from microalgae cells, for example, the biodiesel could be obtained through high temperature and high pressure liquefaction of harvested microalgae, then transesterification of microalgal oil [29]. Although most microalgae had relatively low total lipid content per cell under wastewater conditions, the potentially high biomass productivity would translate to significant total lipid productivity [30, 31]. Microalgae biomass was generally collected by centrifugation or flocculation [32]. In our previous studies, bioflocculant was used to collect the algae cells, and a flocculation efficiency (> 98%) was achieved at both pilot-scale and in situ treatment [33]. The harvesting and reutilization of the microalgae from MPBR is still under investigation in our lab.

## Conclusions

Based on this study, a MPBR operated for algae cultivation showed better performance than a NPBR in terms of both the volumetric microalgae productivity and nutrients removal rate when the real ADW was supplied as the cultivation medium. The better performance of the MPBR was due to the HFM inserted in the reactor, which acted as a solid–liquid separator and thereby enabled the reactor to not only eliminate the inhibitory effects of suspended solid and microorganisms, but also maintain a high microalgae concentration to obtain a high amount of ammonia and phosphate removal. This study offers a valuable case and will advance further research in this field.

## Methods

### Microalgae strains and inoculum production

*Scenedesmus dimorphus* (*S. dimorphus*, FACHB-1266), *Scenedesmus quadricauda* (*S. quadricauda*, FACHB-1297), and *Chlorella sorokiniana* (*C. sorokiniana*, FACHB-275) were purchased from algal-species database of Wuhan Institute of Hydrobiology, Chinese Academy of Sciences. The *Chlorella vulgaris ESP*-*6* (*C. vulgaris ESP*-*6*) used in this study was obtained from the Department of Chemical Engineering, National Cheng Kung University, Taiwan.

The medium used in this study was a modification of BG11 Medium [34]. All selected microalgae species were inoculated in autoclaved BG-11 medium (121 °C, 20 min) in 100 mL Erlenmeyer flasks. At the beginning of each experiment, 60 mL of medium was put into flasks with pre-cultured microalgae. The culture flasks were incubated in a light growth incubator under the optimized conditions at 25 ± 1 °C with an irradiance of 110 μmol^−2^ m^−1^ and light/dark cycles (L:D) of 12:12 h [35]. Periodic agitations were performed for three times each day.

### The source and properties of ADW

The ADW was collected from the biogas project of Nanjing University of Technology, China, which was the anaerobic digestion of pig manure and straw. To avoid variation in its composition, the obtained ADW was immediately stored at 4 °C. The ADW (pH = 8.5 ± 0.5) was a complex mixture with varying chemical properties and contained the following main components in Table 4.

**Table 4:** Biogas slurry nutritional liquid formula

Component	Solution (mg/L)	Component	Solution (mg/L)
TN	1054.4	TP	27.6
NH_4_-N	1024.8 ± 41.6	PO_4_^3−^-P	26.08 ± 2.32
COD	2432.5 ± 241.4	N:P	38:1

### Membrane photobioreactor (MPBR)

The MPBR was designed as shown in Fig. 1. A HFM was inserted into a normal tubular bioreactor. The circulation velocity was set at 150 mL/min. The ADW in the ADW storage chamber flowed through the hollow fiber membrane circularly. The algae in the microalgae cultivation chamber also circulated outside the hollow fiber membrane. Thus, nutrients could permeate through the membrane and be absorbed by the algae, while suspended solids and competitors were trapped in the ADW storage chamber. The MPBR was made of polyvinyl chloride (PVC) and had a height of 30 cm with an external diameter of 5 cm. The hollow fiber membrane was made of polyvinylidene fluoride (PVDF) and had different pore sizes of 0.1, 0.5 to 1 μm. The volume of both the microalgae cultivation chamber and the ADW storage chamber was 1 L. At the bottom of microalgae cultivation chamber of the MPBR, gas distributors were installed to provide bubbles. Mixed gas containing 5% CO_2_ in air was bubbled into the microalgae cultures at an aeration rate of 0.3 vvm. The flow rates of CO_2_ and air were controlled by mass flow meters.

### Microalgae cultivation with ADW

The ADW, which only subsided naturally for 1 h, was diluted and then used as culture medium for microalgae cultivation. Hillebrand pointed out that while N:P ratios exceed 22 indicated P limitation [36]. However, N:P ratios of 38:1 (Table 1) were presented in this study. Extra phosphate was supplemented so that all samples contained the same proper concentration of phosphate. The microalgae was cultivated for 9 days in the MPBR fed with diluted ADW set at 25 °C with an irradiance of 110 μmol^−2^ m^−1^ on a 12:12 h light–dark cycle. The control group was microalgae cultivated directly in a normal photobioreactor fed with diluted ADW. Samples were taken daily and measured in triplicate. The initial inoculum density was set as 0.10 ± 0.01 g/L for all experiments.

During continuous multi-batch cultivations of *C. sorokiniana* with ADW in MPBR under the optimum parameters, the algae cultivation was repeated tri-times to insure that algae cultivation with ADW achieved stability. After 15 days of batch cultivation fished, microalgae medium was centrifuged, then three-quarters of the microalgae biomass were removed from microalgae cultivation chamber. And the treated ADW by microalgae in ADW storage chamber was also replaced with raw ADW. Under the same conditions, the performance of tri-batches cultivations of *C. sorokiniana* in the NPBR as a control was compared with the performance of the MPBR. In NPBR, the ADW was centrifuged and membrane filtrated to remove suspended solids which would influence measure of microalgae biomass.

### Determination of components concentrations in ADW

A 5 mL aliquot of the algae suspension or diluted ADW was collected daily from the bioreactor for nutrients analysis. The samples were centrifuged at 9000 r/min for 10 min, and the supernatant was used to determine the ammonia nitrogen, phosphate, COD and total suspended solids content, following standard methods [37].

Light attenuation curve was determined by measuring the local light intensity (μmol/(m^2^ s)) in microalgae suspension with a waterproof probe of irradiatometer (T-10WsA, Konica Minolta Inc, Japan). The starting point was chosen as the front point of the microalgae cultivation chamber in MPBR and NPBR facing the incident light.

### Microalgae analysis

The culture was centrifuged at 9000 rpm for 10 min at 4 °C, and the supernatant was discarded, followed by rinsing with distilled deionized water. The centrifuged biomass was then freeze-dried and weighed.

An 8 mL sample was centrifuged (15 min at 3600*g*) with the supernatant discarded. The cell sediment was resuspended with distilled deionized water and centrifuged again at 3600*g* for 15 min. Samples for protein analysis were homogenized with 6% trichloroacetic acid, and the precipitated protein was analyzed by a modified Lowry [38] method [39].

### Determination of growth kinetics using logistic equation

A logistic equation is a good choice for explaining the growth curve, as it do not include a substrate term in the description of the entire growth profile of the microorganisms. *X* vs *t* gives a sigmoid variation of *X* as a function of *t*, which may satisfactorily explain the lag, exponential and stationary phase of the culture satisfactory [40]

1 $$\frac{{{\text{d}}y}}{{{\text{d}}t}} = K_{\text{c}} X\left( {1 - \frac{X}{X_{\text{max}}}} \right) $$

where *X* is the dry cell weight (g/L), *X*_max_ is the maximum dry cell weight (g/L), and *K*_c_ is the apparent specific growth rate (day^−1^) for this strain. By integrating and rearranging Eq. (1), it can be written as the following equation:

2 $$ X = \frac{{X_{\text{max} } }}{{1 + \left( {\frac{{X_{\text{max} } }}{{X_{0} }} - 1} \right)e^{{ - K_{\text{c}} t}} }}. $$

This may be further rewritten in the form of Eq. (3) where *X*_max_ is ‘*a*’ and $\left( {\frac{{X_{\text{max} } }}{{X_{0} }} - 1} \right)$ is ‘*b*’. These constants were determined by plotting dry cell weight vs time in MATLAB (ver. 1.1.7.) using curve fitting tool and fitting it with Eq. (3). A confidence interval of 95% was taken into consideration to find the fit

3 $$ y = \frac{a}{{1 + be^{{ - K_{\text{c}} t}} }}. $$

### Statistical analysis

All data were analyzed using Microsoft Excel, Origin and SPSS. Treatment effects were analyzed using one-way ANOVA, and the least significant difference (LSD) multiple range test was used to determine the statistical significance (*P* < 0.05) between pairs with SPSS.

## Abbreviations

ADW: anaerobically digested wastewater
MPBR: membrane photobioreactor
NPBR: normal photobioreactor
COD: chemical oxygen demand
SBBRs: sequencing batch biofilm reactors
SBRs: sequencing batch reactors
HFM: hollow fiber membrane
PVC: polyvinyl chloride
PVDF: polyvinylidene fluoride
LDS: least significant difference
